# 

**Published:** 1849

**Authors:** 


### ARTICLE II.

We send to our subscribers, with this number a statement of their accounts, *advance payment* included for the volume commencing with this number. We hope our bills will be met promptly, as we need the money to "pay the prinrer."

